# Effect of cleaning materials on microtensile bond strength of resin composite to primary dentin contaminated with root canal sealers

**DOI:** 10.1186/s12903-023-03090-z

**Published:** 2023-06-16

**Authors:** Asu Çakir

**Affiliations:** Department of Pediatric Dentistry, Ahmet Keleşoğlu Faculty of Dentistry, Karamanoğlu Mehmetbey University, Yunus Emre Campus, Karaman, Türkiye

**Keywords:** Bond strength, Contaminated dentin, Primary teeth, Root canal filling material

## Abstract

**Background:**

There have been no studies on the bond strength of adhesives with dentin surfaces contaminated with root canal sealers in primary teeth without underlying permanent teeth germs. This study investigated the cleaning materials used for primary tooth dentin contaminated with root canal sealers. The aim was to increase the success rate of root canal treatment in pedodontics clinics and retain the teeth for longer.

**Methods:**

The occlusal enamel layer was removed, followed by the application of root canal sealers (AH Plus or MTA Fillapex) to the dentin and cleaning using different irrigation solutions (saline, NaOCl, and ethanol). The specimens were restored using a self-etch adhesive and composite. Sticks with a thickness of approximately 1 mm were obtained from each sample, and the bond strengths were measured using a microtensile testing device. The interfacial morphology of the bonded space was evaluated using scanning electron microscopy.

**Results:**

The control and AH Plus saline groups had the highest bond strengths. The groups cleaned using ethanol had the lowest bond strengths (*p* < 0.01).

**Conclusion:**

Cleaning the dentin with saline-soaked cotton pellets provided the best bond strengths. Therefore, saline is the most effective material for removing both epoxy resin- and calcium silicate-based root canal sealers from the access cavity.

## Background

Early loss of primary teeth has several physiological, functional, and aesthetic consequences. Pulp canal treatment is often needed to retain primary teeth in the mouth [1]. Root canal filling materials used in primary teeth should resorb in a manner corresponding to the natural resorption of primary tooth roots. However, when permanent tooth germs do not develop, the root canal filling materials in primary teeth do not resorb. Successful permanent root canal filling of primary teeth without permanent tooth germs is an important aim of pedodontics. Nonresorbable gutta-percha and root canal sealers, commonly used in permanent teeth, are also used in primary teeth that lack underlying permanent tooth germs [2]. The prevalence of congenital lack of permanent teeth has been reported as 1.52% [3].

Endodontically treated teeth with adequate coronal structure can be restored with composite resin using a direct technique because of their ability to bond with dentin [4, 5]. Compared to enamel, the bonding of composite resins to dentin depends on its organic content and composition, and on fluid in the tubules [6]. Several other factors also contribute to differences in bond strength between the dentin of permanent and primary teeth. Primary teeth have greater dentin thickness and mineralization, tubule density, and tubule diameters compared to permanent teeth. The dentinal tubules are also curved in permanent teeth but straight in primary teeth [7–9]. In addition to root canal filling, successful endodontic treatment also requires cavity restoration, prevention of bacterial leakage, resistance to occlusal forces, tooth tissue preservation, and long-term maintenance [4, 10, 11]. Coronal microleakage is an important clinical problem, particularly in multirooted primary teeth, because it allows the migration of microorganisms from the pulp chamber to the furcation [12].

The canal sealer is also important for the success of root canal treatment. Tissue tolerance, solubility in solvents, insolubility in oral fluids, bacteriostatic properties, and biocompatibility in case of leakage into periapical tissues contribute to the clinical success of root canal sealers [13, 14]. AH Plus (Dentsply DeTrey Gmbh, Konstanz, Germany), an epoxy resin-based sealer, is the gold standard root canal sealer because of its excellent physicochemical properties [15, 16]. MTA Fillapex (Angelus, Londrina, Brazil), a hydrophilic calcium silicate-based canal sealer, is preferred because of its ease of use, biocompatibility, and bioactive properties [17, 18].

Our literature review showed that the bond strength of permanent filling materials, applied after the root canal sealer was wiped from the coronal dentin using cotton pellets soaked in irrigation solutions, in primary teeth without permanent successors has not been studied. The aim was to increase the success rate of root canal treatment in pedodontics, retain the teeth for longer, and maintain chewing function until growth was completed. Therefore, the present study investigated the strength of the resin-dentin microtensile bond after cleaning primary teeth contaminated with AH Plus and MTA Fillapex root canal sealer using various irrigation solutions (saline, 2.5% sodium hypochlorite (NaOCl), 95% ethanol). The null hypothesis was that the irrigation solutions do not affect the dentin bond strength of adhesive system in primary teeth.

## Materials and methods

### Sample size calculation

Based on a power of 85.44%, error of 0.05, and estimated effect size of 0.25, the sample size (the number of test beams) was determined as 30 for one-way analysis of variance (ANOVA), performed using the G*Power software.

### Sample source

We used 36 caries-free primary second molars, extracted due to excessive mobility because of root resorption. The samples were stored in 0.1% thymol solution at 4 °C no longer than 1 month. The samples were obtained from patients referred to the Department of Pediatric Dentistry.

### Inclusion and exclusion criteria

Caries-free primary second molars of healthy individuals, who presented to the clinic with chewing difficulty because of excessive mobility, were included in the study. Carious and filled primary teeth were not excluded.

### Dentin surface preparation and sealer contamination

The occlusal enamel of the primary teeth, stored in 0.1% thymol solution at 4 °C, was removed using a low-speed, water-cooled rotary device (Isomet Low Speed Saw; Buehler Ltd., Lake Bluff, IL, USA) at the Selcuk University Faculty of Dentistry Research Laboratory. The materials used in this study are listed in Table 1.

**Table 1:** Materials used in this study

Materials		Composition	Application mode
Root canal sealers	MTA Fillapex	Salicylate resin, diluting resin, natural resin, bismuth trioxide, nanoparticulated silica, MTA, pigments	It was applied to the dentine with the help of dry cotton pellets
	AH Plus	A paste: Epoxy resin B paste: amine derivatives	It was applied to the dentine with the help of dry cotton pellets
Irrigations	NaOCI	2.5% in intensity	It is applied with the help of cotton pellets
	Ethanol	95% in intensity	It is applied with the help of cotton pellets
Bonding	Prime & Bond Universal; Dentsply, Germany	Diamine Bis Acrylic; Water; Propanol; Dihydrogen Phosphate Methacrylate; Penta; Bis Acrylic Propylamine; Camphorquinone; HexaFluorPhosphate; Benzonitrile Dimethylamino; Hydroquinone	Bond was applied and waited for 20 s, the air was dried with water spray for 5 s and light was applied for 10 s
Composite	Nova Compo C	Dimetakrilat, ba-glass, yiterbiyum triflorur, prepolimerized filler, catalyst, stabilizer, ULS monomer	Resin composite was applied and light was applied for 20 s

The teeth were washed, dried using an air–water spray, and randomly divided into three groups.

- Group 1 (Control group): Root canal sealer and irrigation solution were not applied.
- Group 2: AH Plus sealer (AH Plus; Dentsply) was applied to the dentin surface using a dry cotton pellet for 5 min.
- Group 3: MTA Fillapex sealer (MTA-Fillapex; Angelus) was applied to the dentin surface using a dry cotton pellet for 5 min.

The root canal sealer was applied once to cover the entire dentin surface. Groups 2 and 3 were further divided into three subgroups based on the irrigation solution.

- Subgroup a: Contaminated dentin was cleaned for 1 min using cotton pellets soaked in saline.
- Subgroup b: Contaminated dentin was cleaned for 1 min using cotton pellets soaked in 2.5% sodium hypochlorite (NaOCl).
- Subgroup c: Contaminated dentin was cleaned for 1 min using cotton pellets soaked in 95% ethanol.

The samples were examined under a light microscope to ensure that there were no cotton pellet fibers on the application surface prior to scanning electron microscopy (SEM).

### Bonding and restoration

After sealer removal, the teeth were restored using self-etch adhesive (Prime & Bond Universal; Dentsply) and composite (Nova Compo C Composite; Imicryl Corp., Konya, Turkey) according to the manufacturer’s instructions. The enamel edges were etched before adhesive application. The dentin was then etched, and the acid gel was removed and rinsed. Prime & Bond Universal was applied to the entire cavity surface, taking care to avoid pooling, and was agitated slightly for 20 s. The adhesive was light-cured for 10 s, and the solvent was air-evaporated for at least 5 s. A 2-mm-thick composite restoration was applied to the bonded dentin surface using the incremental technique, and cured using an LED curing unit (VALO Cordless; Ultradent Products Inc., South Jordan, UT, USA) according to the manufacturer’s instructions.

### Sample preparation and microtensile testing

After polymerization, the specimens were stored in distilled water at 37 °C for 24 h. Specimens were then sectioned into multiple 1 × 1-mm beams using the Isomet Low Speed Saw under water cooling for the “non-trimming” version of the microtensile test. After excluding external beams from the periphery, seven or eight central beams were randomly selected from each tooth-composite specimen. Thirty beams were obtained from each group for microtensile bond strength (MTBS) testing (*n* = 30). Beams from the peripheral dentin were excluded from the study, and pre-test failures were excluded from the statistical analysis. The beams were stored in distilled water at 37 °C until the MTBS test.

The beams were fixed to a universal testing machine (Microtensile Tester; Bisco, Schaumburg, IL, USA) using cyanoacrylate glue (Akfix, Istanbul, Turkey), and were stressed at a cross-head speed of 1 mm/min until failure. The cross-sectional area at the site of failure was measured to the nearest 0.01 mm using a digital caliper (Starret 727–6/150; Starret, San Paulo, Brazil), and the MTBS was calculated and expressed in MPa.

One-way ANOVA was used to analyze differences between groups, and Tukey’s multiple comparison test was used to compare group means (*p* = 0.05).

The failure modes for all specimens were evaluated using SEM at 100 × magnification (EVO LS10; Zeiss, Oberkochen, Germany). Failures were classified as adhesive (between dentin and resin), cohesive (within the resin or dentin), or mixed (combination of adhesive and cohesive failures).

## Results

Pre-testing failures were recorded for both the sealers, regardless of whether they were cleaned using saline, NaOCl, or ethanol (two, three and five failures for MTA and one, two and four failures for AH Plus, respectively). The MTBS values, standard deviations, and failure types are summarized in Table 2.

**Table 2:** Average and standard error values and failure types of applications (MPa) (*n* = 30)

Application	$\overline{X}\pm {S }_{\overline{X} }$	Failure Type (Adhesive %)	Failure Type (Mixed %)	Failure Type (Cohesive %)
Control	25.93 ± 1.289^A^	56	44	0
AH Plus Saline	25.37 ± 1.229^A^	49	51	0
MTA Fillapex Saline	23.98 ± 0.993^B^	53	47	0
AH Plus NaOCl	17.07 ± 0.932^C^	33	67	0
MTA Fillapex NaOCl	15.23 ± 2.651^D^	46	54	0
MTA Fillapex Ethanol	8.00 ± 0.429^E^	66	34	0
AH Plus Ethanol	5.39 ± 0.592^F^	80	20	0

Within the column, values indicated by different capital letters showed statistically significant differences between materials.(A-H:*p* < 0.01)

On ANOVA, the differences between the means of the control and AH Plus wet cotton groups were not statistically significant (*p* > 0.01). However, the difference between the remaining groups was statistically significant (*p* < 0.01) (Table 2). The control and AH Plus saline groups had the highest MTBS values. Compared to the control group, the ethanol subgroups for both sealers had lower bond strengths (*p* < 0.01).

**Fig. 1:**
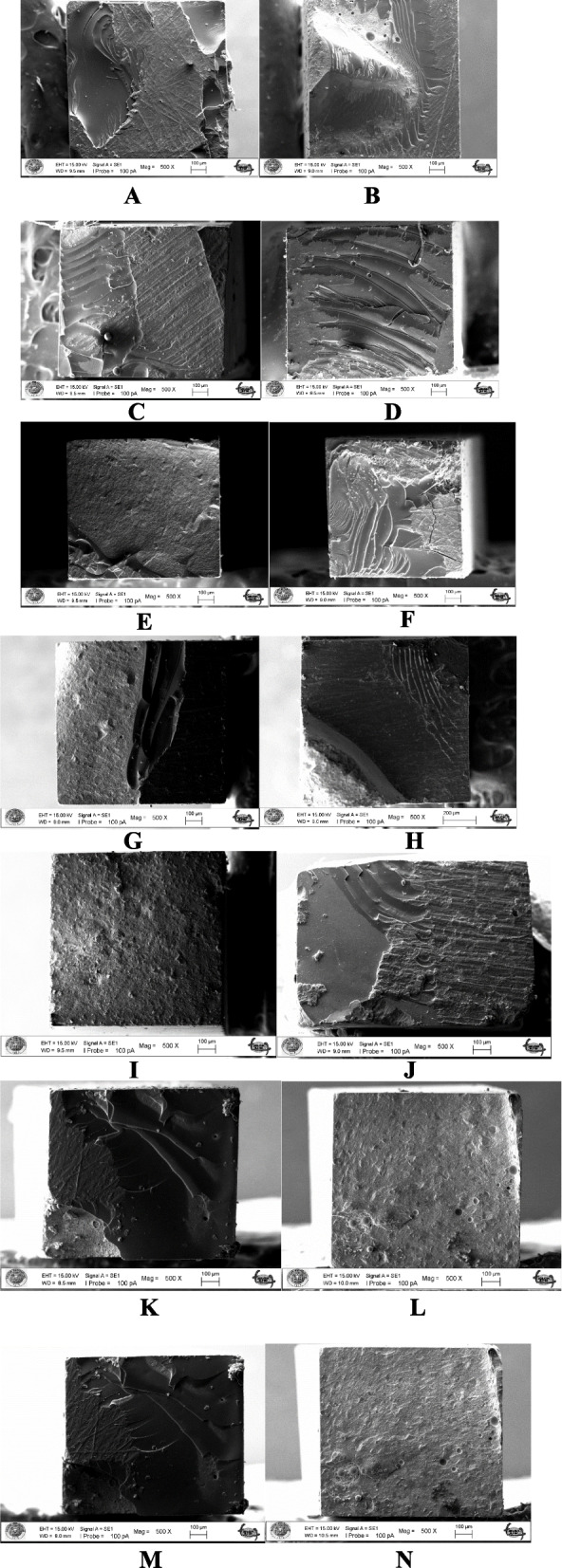
Composite/dentin failure types of samples at 100 × magnification by SEM. **A**, **B** adhesive failured of control groups; **C**, **D** mixed and adhesive failured of AH Plus saline; **E**, **F** adhesive failured of AH Plus NaOCl; **G**, **H** adhesive and mixed failured of AH Plus ethanol; **I**, **J** adhesive and mixed failured of MTA Fillapex saline; **K**, **L** adhesive failured of MTA Fillapex NaOCl; **M**, **N** adhesive failured of MTA Fillapex ethanol. Legend: SEM, scanning electron microscope; adhesive failures, between dentin and resin; cohesive failures, within the resin or dentin; mixed failures, combination of adhesive and cohesive failures

**Fig. 2:**
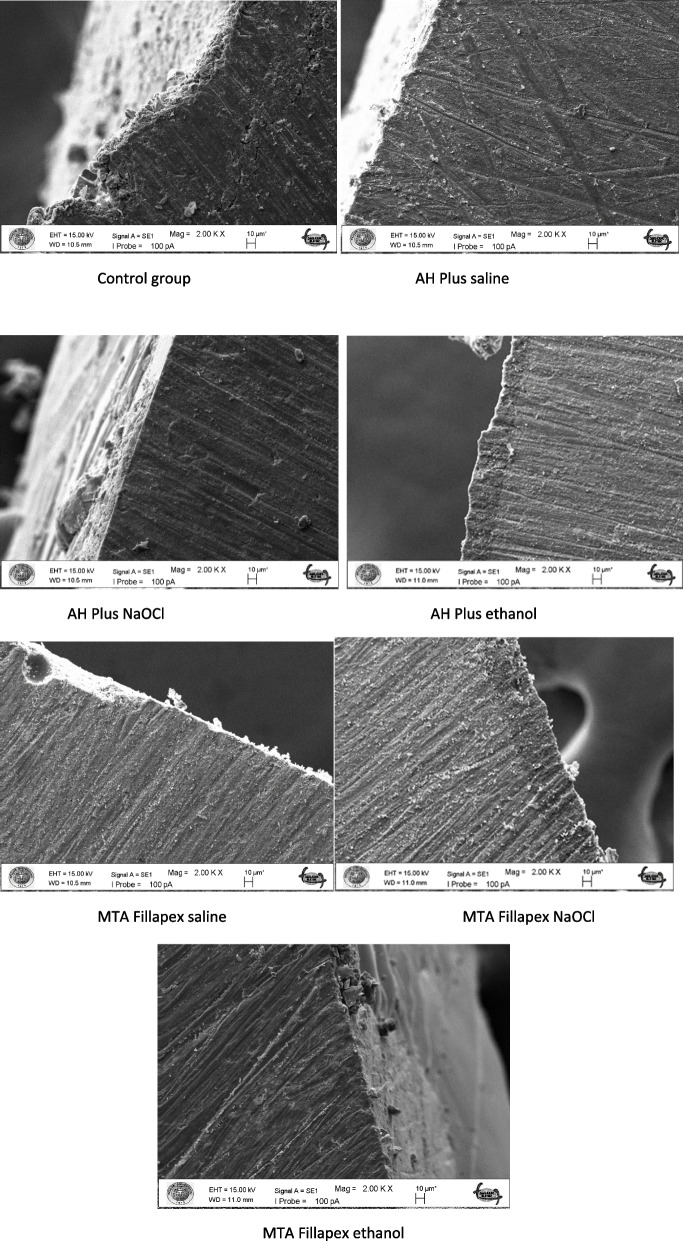
Scanning Electron Microscope (SEM) Longitudinal images of the failure type of each group

Failure-type analysis using SEM demonstrated the presence of adhesive and mixed failures in the samples (Figs. 1 and 2, Table 2).

## Discussion

It has been widely reported that coronal microleakage and weak bond strength reduce the success of root canal treatment [19, 20]. However, the sealer may also affect the bond between dentin and the materials used during root canal obturation, thus compromising the seal [21]. The present study demonstrated that the solutions used for cleaning the dentin contaminated with canal sealers can affect the bond strength of restorative systems.

The effects of root canal filling materials and pretreatment with solvents on the shear bond strength of composite resin in primary tooth dentin has been investigated previously. Cleaning the samples with 96% ethanol after removing Metapex was found to significantly increase the composite-dentin bond strength [22]. Ethanol is routinely used as a solvent for surface cleaning because of its easy availability, but its effectiveness was found to be unfavorable [23–25]. Another study reported that ethanol was effective in reducing the negative effects of root canal filling materials on the adhesion of primary tooth dentin [26]. In the present study, the bond strength for teeth contaminated with canal filling sealers and cleaned using ethanol was significantly lower compared to the other groups.

NaOCl is frequently used in endodontic treatment, and studies have shown that it reduces the bond strength between adhesive materials and dentin by causing changes in the dentinal collagen fibrils [5, 27, 28]. In the present study, the MTBS values for the samples cleaned using NaOCl were higher than those for the ethanol groups, but significantly lower than those for the control and saline groups. Therefore, the use of saline may promote clinical success because it is cheap, safe, easily available, and non-irritant. The difference between the groups treated with MTA Fillapex-NaOCl and AH Plus-NaOCl was also significant.

In consideration of the negative effects of chemical solvents on adhesion, Kürklü et al. used water to clean dentin contaminated with bioceramic-based sealers, and found that the elimination of canal sealer from the access cavity was related to the amount of water used [29]. The AH Plus sealer used in the present study is hydrophobic, unlike other endodontic canal sealers [30]. Borges et al. (2014) examined the physicochemical properties of MTA Fillapex and AH Plus, and found that MTA Fillapex had a more homogeneous appearance and higher resolution [31]. In another study, the water solubility of bioceramic sealers was found to be higher than that of resin-based sealers [32]. MTA Fillapex dissociates into calcium hydroxide, and subsequently into calcium and hydroxyl ions, when it contacts water, which increases the pH of the solution [33]. A high pH prevents the destruction of mineralized tissue by neutralizing the acids secreted by osteoclasts [16]. However, in the present study, the MTBS values for AH Plus cleaned with wet cotton were higher than those for MTA Fillapex.

Like previous studies of dentin contaminated with canal sealers, flat surface dentin was used in the present study to assess the bond strengths [23, 28, 34]. Additionally, coronal dentine was used instead of pulp chamber dentine due to the difficulty of acquiring a consistent dentine surface for testing. Flat dentin surfaces are easier to clean and ensure the complete removal of residual materials and cotton from the surface. However, the flat dentin surface does not exactly imitate the cavity conditions used in the clinic. Most recent studies of canal sealer removal used dry cotton pellets and organic solvents. It has been reported that cleaning with dry cotton pellets reduces the bond strength to 69–88% [23, 34, 35]. In the present study, the samples were examined under a light microscope to ensure that there were no residual cotton pellet fibers on the application surfaces at the time of SEM examination.

In the current study, failures in the samples were predominantly adhesive, which was consistent with previous studies. Goncalves et al. investigated the effects of different irrigation protocols with a methacrylate-based endodontic sealer on the radicular dentin interface and ligament strength, and reported predominantly adhesive failures in all groups [36]. Prado et al. also investigated the effects of different irrigation protocols on the dentin and resin sealer bond strengths, and found that the failures were mainly adhesive [37].

Our literature review showed that the bond strength of permanent filling materials, applied after the root canal sealer was wiped from the coronal dentin using cotton pellets soaked in irrigation solutions, in primary teeth without permanent successors has not been studied. Most studies about surface treatment techniques to improve the adhesion are limited to permanent teeth [38]. Hence, assessing differences in previous studies was difficult. Additional studies are required to support the results of this study.

In the present in vitro study, the bond strengths of primary tooth dentin, contaminated with canal sealers, varied with the cleaning materials. The findings of the present study may increase the clinical success rate of root canal treatment in primary teeth without permanent successors. Further studies are needed to validate these findings. However studies on primary teeth with different canal filling materials and cleaning protocols may led to new perspectives in the field of pedodontics. The present study only tested immediate bond strengths using a single type of composite and bonding. Future studies with sample aging and long-term bond strength parameters using different bonding and composites are required to confirm these results.

## Conclusions

Materials used to remove root canal sealers can affect the adhesive system bond strength dentin. Our saline group can be suitable cleaner for both epoxy resin- and calcium silicate-based root canal sealers. NaOCl and ethanol negatively affected the bond strength. However, ethanol had the most significant effect (*p* < 0.05).

## Data Availability

The datasets used and analysed during the current study are available from the corresponding author on reasonable request.
